# Climate change and cancer: converging policies

**DOI:** 10.1002/1878-0261.12781

**Published:** 2020-09-22

**Authors:** Paolo Vineis, Inge Huybrechts, Christopher Millett, Elisabete Weiderpass

**Affiliations:** ^1^ Grantham Institute for Climate Change and School of Public Health Imperial College London UK; ^2^ International Agency for Research on Cancer Lyon France; ^3^ School of Public Health Imperial College London UK

**Keywords:** air pollution, biodiversity, climate change, cobenefits, externalities, ultraprocessed food

## Abstract

Intervening on risk factors for noncommunicable diseases (including cancer) in industrialized countries could achieve a reduction of between 30% and 40% of premature deaths. In the meantime, the need to intervene against the threat of climate change has become obvious. CO_2_ emissions must be reduced by 45% by the year 2030 and to zero by 2050 according to recent agreements. We propose an approach in which interventions are designed to prevent diseases and jointly mitigate climate change, the so‐called cobenefits. The present article describes some examples of how climate change mitigation and cancer prevention could go hand in hand: tobacco control, food production, and transportation (air pollution). Many others can be identified. The advantage of the proposed approach is that both long‐term (climate) and short‐term (health) benefits can be accrued with appropriate intersectoral policies.

## Graphical abstract

Many human activities cause a double burden, on health and on the environment. A few examples are as follows: Air pollution causes greenhouse gas emissions and several human diseases (left). Food production and trades are involved in loss of biodiversity, greenhouse gas emissions, and human diseases such as zoonoses or excess cancer and cardiovascular diseases (right). Intersectoral policies can contribute to the achievement of several Sustainable Development Goals.

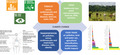

**Abbreviations**
GHG: greenhouse gases
IARC: International Agency for Research on Cancer
LMICs: low‐ and middle‐income countries
NCD: noncommunicable disease
PMI: Philip Morris International
SDGs: Sustainable Development Goals
UPF: ultraprocessed food

## 1 Introduction

Apparently, the global epidemic of cancer [1] and the threat of climate change [2] have little to do with each other. Climate change is a reality whose broad planetary implications have started to be investigated recently and include health effects like the spread of infectious diseases and deaths from heat waves [2]. Noncommunicable diseases (NCDs) including cancer are becoming a global problem, with 18 million new cancer cases estimated in 2018 and growing rates in low‐ and middle‐income countries (LMIC; https://gco.iarc.fr/). At a second look, there is much overlap between climate change and NCD both on scientific grounds and in the development of policies. If we examine the main causes of cancer as listed among ‘Group 1’ carcinogenic agents by the International Agency for Research on Cancer (IARC) Monographs program (i.e., carcinogenic to humans, see https://monographs.iarc.fr/iarc‐monographs‐preamble‐preamble‐to‐the‐iarc‐monographs/), several of them are also associated with a non‐negligible planetary footprint.

**Fig. 1:**
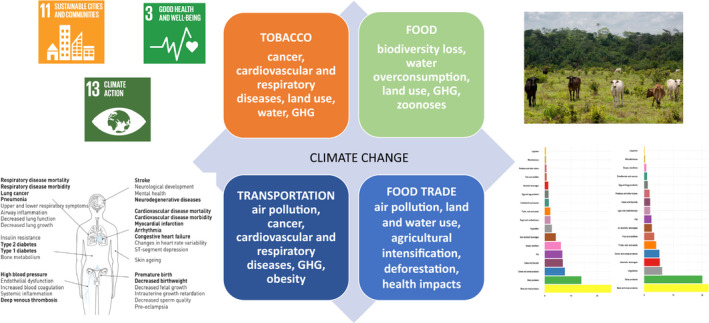
Many human activities cause a double burden, on health and on the environment. A few examples are as follows: Air pollution causes GHG emissions and several human diseases (left). Food production and trades are involved in loss of biodiversity, GHG emissions, and human diseases such as zoonoses or excess cancer and cardiovascular diseases (right). Intersectoral policies can contribute to the achievement of several SDGs.

In this article, we identify gaps in knowledge that require further research and policy investments. We provide examples to highlight how the multiple externalities of different economic activities need to be fully mapped. Externalities are the consequence of activities that affect other parties without this being reflected in market prices, and include effects on the health of the populations and planetary effects such as climate change, deforestation, and land and water use. Currently, such a map is largely incomplete. The figure exemplifies some of the externalities of different commodities or activities, having an impact on both human health and the planetary footprint. The identification of externalities should lead to the development of intersectoral policies that respond to the concept of ‘cobenefits’, that is, policies that contribute to the prevention of NCDs (including cancer) and of environmental damage including climate change (Fig. 1).

## 2 Planetary externalities of behavioral or environmental carcinogens

Let us start with perhaps the least obvious, tobacco. Tobacco is notoriously responsible for about one‐third of cancer deaths in the world, in addition to cardiovascular, respiratory, and other diseases [3], but it also has a considerable planetary footprint. Data on emissions have been provided by Philip Morris International (PMI) and summarized in the only paper available so far [4]. Direct emissions from PMI in 2017 were 229 116 tons of CO_2_ equivalents from manufacturing and 118 487 tons due to the vehicle fleet. Indirect emissions (for energy use) were 434 460 tons of CO_2_ equivalents from manufacturing and 15 800 tons from offices. Emissions of greenhouse gases (CHGs) incorporated in purchased goods and services, transportation, and distribution amounted to 3 611 000 tons, that is, the vast majority [4]. The sum of these emissions, only for one manufacturer and 1 year, is more than 4.5 million tons, not a negligible amount. Tobacco manufacturing is also extremely water‐use‐intensive with at least 23 247 thousands of cubic meters per year. According to the review [4], should tobacco companies incorporate environmental externalities (water use, air pollution, land degradation, etc.) in their costs, tobacco would not be a profitable industry. As opposed to food, tobacco smoking is not a human necessity.

Next, and overall comparable to tobacco for their negative impact on health, are unhealthy diet and obesity [3]. Reducing our meat consumption could help prevent a range of NCDs and infectious diseases. An IARC Monograph Working Group has categorized processed meat as carcinogenic to humans, and red meat as probably carcinogenic [5]. Also, infectious disease risks are influenced by food production and supply choices. For example, in the United States, food‐borne infections that result in hospitalization or death, such as from *Campylobacter*, *Clostridium*, *Listeria,* and *Salmonella*, are more often associated with animal‐based foods (particularly poultry) than vegetable‐based foods. And also in the case of meat consumption, the planetary footprint is huge. Livestock production for animal‐based food is immense, with around 3.5 poultry and 0.5 red meat mammals raised per year for every one of over seven billion people (https://www.statista.com/statistics/263962/number‐of‐chickens‐worldwide‐since‐1990/). This growth in livestock production has had severe impacts on natural ecosystems, ranging from land degradation to major consequences for biodiversity (e.g., livestock production negatively impacts wildlife due to competition for resources). More importantly, meat production is a major source of GHG emissions. Agriculture has been estimated to contribute at least 11‐14% or ~ 5.0–5.8 GtCO_2_e (giga‐tonnes of equivalent carbon dioxide) per year of total anthropogenic GHG emissions (but other, larger figures have been reported), of which ~ 75% is produced through the livestock sector [6]. These estimates are subject to uncertainties, depending on methodologies and assumptions used in their assessments. The main GHG emitted in the livestock sector is methane, which accounts for 44% of all emissions from this sector. Also, more than a third of the food sector's global water use is through the livestock and dairy industries alone.

Lower nutritional quality of diets is overall associated with higher planetary footprint [7]. There are several more environmentally friendly sources of protein that could replace meat, also reducing the water footprint of meat protein. There have been several proposals to develop dietary advice that may have a positive impact on both human health and planetary health, such as the EAT‐Lancet diet ‘for the Anthropocene’ [7]. This particular diet was developed to ensure—among other measures—the achievement of Paris Agreement temperature rises. The EAT‐Lancet diet could avoid ~ 11.1 million deaths per year (in 2030) in the world and reduce premature mortality by 19% [7].

Another area of overlap between ill health, including cancer, and climate change is air pollution. An IARC Monograph Working Group concluded that outdoor air pollution is carcinogenic to humans (sufficient evidence), with particular focus on particulates [8]. Air pollutants associated with fossil fuel combustion have other well‐documented adverse human health effects beyond cancer (e.g., cardiovascular and respiratory diseases). Changes in transportation, in particular an increase in the promotion of active transportation such as walking and use of bicycles, may reduce air pollution, while at the same time contributing to better health in several ways, by increasing physical activity and, thus, reducing the risk of obesity, diabetes, cancer, and cardiovascular disease. As an example, it has been estimated that clean energy policies in the United States could prevent 175 000 premature deaths by 2030 and 22 000 annually thereafter, and clean transportation could prevent 120 000 US premature deaths by 2030 and about 14 000 annually thereafter [9]. But, once again, the emission of air pollutants has also an important impact on climate: transportation overall contributes to 13% of all GHG.

A commitment to including health and climate in all policies should be taken seriously by all institutions, in the context of the attainment of the Sustainable Development Goals (SDGs) [10, 11].

## 3 International trades of food and their planetary and health impact

Nobody has estimated yet the overall impact of international trade of food on planetary and human health. Such impact includes, for example, air pollution related to transportation, the ensuing climate changes, and land and water use in low‐income countries. Agricultural land use and land‐use changes, including agricultural intensification, deforestation, and the conversion of wildland into crops and pasture, have led to major ecological consequences. The potential impacts on human health of such massive changes are only partially known and have mainly focused on occupational pesticide, chemical, and heavy metal exposure (some of which have been found to be carcinogenic to humans). However, there is increasing evidence that links human‐induced land use to infectious disease risk, including exposure to carcinogenic parasites. Rohr *et al*. [12] report that agricultural drivers are associated with > 25% of emerging infectious diseases and > 50% of emerging zoonotic infectious diseases in humans. In a recent systematic review, Shah *et al*. [13] quantified the association between where people live or work in South‐East Asia and disease risk. They found that those living on agricultural land were on average almost twice as likely to be infected with a pathogen as controls. There were also consistent associations between forest monoculture agriculture (palm oil and rubber) and a number of specific diseases. From the point of view of cancer, it is worth noting that some of the infectious agents involved in these changes are carcinogenic, including *Opistorchis viverrini* and *Clonorchis sinensis* (liver flukes), both group 1 carcinogenic agents according to an IARC Working Group [14]. Changes in habitats and in global and local climate may lead to the spread of these agents beyond their current borders, as is the case for *Schistosoma* ssp in China, though there are many knowledge gaps [15].

## 4 Industrialized processing of food, loss of biodiversity, and cancer

Industrialized processing of food or ‘ultraprocessing’ has grown rapidly in Europe and globally since the 1970s. The purpose of ultraprocessing is ‘to create branded, convenient (durable, ready to consume), attractive (hyper‐palatable) and highly profitable (low cost ingredients) food products designed to displace all other food groups’ [16]. Recent studies indicate that 55% and 60% of total calories consumed in the United Kingdom and the United States, respectively, are from ultraprocessed foods (UPFs) [17, 18] and that growth in their consumption is now increasing most rapidly in LMICs.

International studies have shown that when compared with minimally processed foods and freshly prepared meals, UPFs have higher energy density, higher content in free sugars and salt, saturated and trans‐fats, and lower fiber and micronutrient content [17, 19]. The worldwide shift toward a dramatic increase in the consumption of UPFs appears partly responsible for the global obesity epidemic [20] and may contribute to an increased risk of cardiometabolic diseases [21]. UPFs might additionally increase the risk of cancer, but there remains limited published data on associated risks [22]. Packaging of UPFs has also been postulated to contain compounds with carcinogenic and endocrine disruptor properties, such as bisphenol A. Furthermore, UPFs contain authorized, but often controversial, food additives such as sodium nitrite in processed meat for which carcinogenicity has been suggested in animal or cellular models. Just like tobacco use, some of the UPFs (e.g., distilled spirits) may have a significant impact on human health and our environment, while not being a human necessity.

One of the major negative impacts of UPF production, related to agriculture intensification, is on biodiversity. Biodiversity is the term used to describe the variability among living organisms from all sources, including diversity within species, between species, and of ecosystems. In turn, food biodiversity—the diversity of plants, animals, and other organisms used for food, both cultivated and from the wild—is a critical element in response to global malnutrition, and it supports sustainable food systems. Biodiversity is relevant to SDGs and to the concept of planetary boundaries as developed by Rockstrom *et al*. [23]. The SDG 2 is ‘End hunger, achieve food security and improved nutrition and promote sustainable agriculture’, while one of the nine key environmental boundaries that humanity must stay within in order to keep the planet hospitable is biospheric integrity (the opposite of biodiversity loss and extinctions). In fact, one of the biggest threats to biodiversity is food systems and agricultural intensification. Clearing uncultivated land for farming can lead to the destruction of natural ecosystems, with massive effects on the local wildlife and biodiversity. Converting wild to domesticated species, monoculture, chemical pollution, and loss of biomass, all threaten the world's ability to sustain life of all kinds.

Concerning human health, promoting a diversity of foods in human diets, in particular a variety of distinct edible species, has potential cobenefits from both a public health and a sustainable food system perspective. Food biodiversity provides the necessary nutrients and is an essential component of local food systems, cultures, and food security. Human diets that used to be composed of a wide variety of plants and animals have gradually shifted to a diet composed of mostly processed foods and comprising a limited number of species. While an estimated 300 000 edible plant species are available to humans, more than half of the global energy need is currently met by only four crops: rice, potatoes, wheat, and maize [24].

Sustainable diets were previously defined as ‘those diets with low environmental impacts which contribute to food and nutrition security and to healthy life for present and future generations’ [25].

From a conservation point of view, diets based on a wide variety of species have lower pressure on single species. Increased species diversity, in turn, is associated with increased stability and resilience, and enhanced productivity of natural and agricultural ecosystems [26]. Several countries, including Brazil, Sweden, Qatar, and Germany, have expressed concerns regarding sustainability of diets in food‐based dietary guidelines [27], and the Nordic Nutrition Recommendations have specific considerations for biodiversity in human diets [28].

Several observational studies have shown that consumption of different food groups is inversely associated with colorectal cancer risk [22] and all‐cause mortality [29]. To date, however, the evidence regarding the potential health benefits of food biodiversity (e.g., species richness) in human diets is still scarce. In addition, observational studies investigating cobenefits of higher food biodiversity of our diets for human and planetary health are still lacking.

## 5 Conclusions

Intervening on risk factors for NCDs (including cancer) in industrialized countries could realistically achieve a reduction of between 30% and 40% of premature deaths from NCD (https://www.who.int/news‐room/factsheets/detail/noncommunicable‐diseases), including cancer. The risk factors include tobacco use, unhealthy diets, and low levels of physical activity (http://www.healthdata.org/sites/default/files/files/country_profiles/GBD/ihme_gbd_country_report_

italy.pdf). They also include the impact of environmental exposures, particularly of air pollution. Considering that NCDs, including cancer, represent 70% of all causes of mortality, there are plenty of opportunities for prevention, resulting in prolonged years of life in good health and a reduction in healthcare costs.

The need to intervene against the threat of climate change is now obvious. Based upon the Paris Agreement and the subsequent Katowice meeting, CO2 emissions must be reduced by 45% by the year 2030 and to zero by 2050. Given that action against climate change is primarily taken via energy choices, limiting the use of fossil fuels, and promoting renewable sources, a very effective tool is one in which interventions are designed to prevent diseases and jointly mitigate climate change, the so‐called cobenefits. When choosing climate change mitigation actions, it is extremely important to bear in mind their effect on health [10]. For example, there are numerous compounds emitted into the atmosphere that contribute to climate change: carbon dioxide (CO_2_), carbon, nitrogen oxides, and fluorinated gases (to name a few), some of which also have consequences on health. If mitigation policies focussed only on carbon dioxide, one would lose the positive effects on health that would arise from broader actions. Policies based solely on carbon capture and storage would not be accompanied by all the benefits of eliminating the other polluting derivatives resulting from the combustion of coal and petroleum, including particulate matter, polycyclic aromatic hydrocarbons, heavy metals, and others. This is only an example of the need to consider all externalities of human technologies and of considering remediating policies that address jointly climate change and health.

## Author contributions

All authors contributed equally.

## Conflict of interest

The authors declare no conflict of interest. Where authors are identified as personnel of the International Agency for Research on Cancer/World Health Organization, the authors alone are responsible for the views expressed in this article and they do not necessarily represent the decisions, policy or views of the International Agency for Research on Cancer/World Health Organization.
